# Combined photographic and ultrasonographic measurement of the ANB angle: a pilot study

**DOI:** 10.1007/s11282-017-0275-y

**Published:** 2017-03-21

**Authors:** Alberto Di Blasio, Chiara Di Blasio, Giuseppe Pedrazzi, Diana Cassi, Marisabel Magnifico, Edoardo Manfredi, Mauro Gandolfini

**Affiliations:** 1 S.Bi.Bi.T. Department, University of Parma (Italy), Via Gramsci 14, 43126 Parma, Italy; 2 Head and Neck Department, University of Parma (Italy), Via Gramsci 14, 43126 Parma, Italy; 3 Doctoral School in Life and Health Science, University of Parma (Italy), Via Gramsci 14, 43126 Parma, Italy; 4 Neuroscience Department, University of Parma (Italy), Via Volturno 39, 43125 Parma, Italy

**Keywords:** Orthodontics, Cephalometry, Ultrasonography, Dimensional measurement accuracy, Radiation protection

## Abstract

**Objective:**

This study was performed to evaluate the feasibility of noninvasive measurement of the ANB angle using photographic and ultrasonographic methods.

**Methods:**

Twenty consecutive orthodontic patients were evaluated. The ANB angle and soft tissue thickness covering the N, A, and B cephalometric points were measured by lateral teleradiography; these measurements were made by two expert operators. The soft tissue thickness covering the N, A, and B cephalometric points was measured by ultrasonography; these measurements were also made by two expert operators. On a 1:1 photographic profile print on which the ultrasonographic points were marked, the ANB ultrasonographic angle was measured. The following comparisons were considered: averaged and single measurements of N, A, and B points by first versus second ultrasonographer; averaged and single ultrasonographic versus radiographic soft tissue thickness covering the N, A, B points; and averaged and single ultrasonographic versus radiographic measurements of ANB angle.

**Results:**

High correlation and concordance of the averaged and single measurements, but no significant difference, was found between the two ultrasonographers. No statistically significant difference was found between the two methods for measuring averaged soft tissue thickness, but a 20% difference was found for the single measurements. High correlation and concordance between the ultrasonographic and radiographic measurements, but no significant difference, was found between the single and averaged ANB angle measurements.

**Conclusion:**

Ultrasonography seems to be a noninvasive and reliable technique for measurement of the ANB angle and may replace radiographic measurement in some cases.

## Introduction

Although some controversy regarding the correct use of lateral cephalometric radiographs is still present in orthodontic textbooks, cephalometric analysis is the basis of every type of orthodontic treatment planning [1–3]. For most orthodontists, cephalometric radiography is the standard imaging technique and an invaluable means of obtaining diagnostic information for the management of malocclusion and skeletal disharmony. Cephalometric radiographs, introduced to the field of orthodontics by Broadbent [4, 5] in 1931 in the US, were soon employed by early investigators [6–9] to assess the skeletal relations of the facial bones and the long-axis inclination of the anterior teeth. In 1953, Steiner [10–12] proposed his original analysis containing a description of the ANB angle. This angle relates the anterior limit of the maxillary bone (A point) and mandibular bone (B point) with the anterior limit of the nasofrontal suture (N point). The ANB angle measures the relative anteroposterior position between the maxilla and mandible. In normal individuals, the ANB angle is 2° ± 2° at the end of growth. Since its first description in 1953, the ANB angle has remained one of the most frequently measured cephalometric data to assess the maxillomandibular relations, even in complex cases involving orthodontic or orthognathic surgery [13–16]. From 1950 to 1970, the progress in orthodontic cephalometry was logarithmic, and a large number of analyses were proposed. The golden age of cephalometry ended in 1970 with the complex analysis proposed by Delaire [17–19] and Delaire et al. [20]. Today, orthodontists are able to measure the proportions of the human face with a high degree of precision using a very wide range of different cephalometric analyses. Not every orthodontist uses the same analysis in clinical practice; each orthodontist selects the technique that best meets his or her needs and expectations. Despite these differences, all clinicians agree that cephalometry is an unavoidable step in orthodontic treatment planning. However, concerns regarding radiographic exposure, particularly in growing individuals, may limit its use [21]. This is especially problematic because the use of longitudinal radiographs to assess a patient’s growth and therapeutic outcome is still a common practice [22]. A new radiographic technique may only be advised when its outcome results in a different treatment decision. Other clinical analysis techniques such as anthropometry may be used to avoid frequent radiographic exposure and may be useful in further understanding the patient’s structure. Using anthropometrics, the orthodontist directly examines the patient’s face or facial photographs to understand the deformity and appreciate the progressive effect of the therapy [23]. Several authors have proposed anthropometric evaluations, sometimes creating a very complex analysis, as in Arnett’s soft tissue cephalometric analysis [24–28]. Unfortunately, this clinic facial evaluation cannot fully replace cephalometry because skeletal orthodontic therapy is indicated in growing patients while facial anthropometry has only been well studied in adults, and not every face presents the same soft tissues thickness covering important points such as the N, A, and B points. Another way to perform noninvasive evaluation of the facial structure, the DigiGraph work station, was described in 1990 by Chaconas et al. [29, 30], in 1995 by Prawat et al. [31], and in 1999 by Tsang and Cooke [32]. Despite the good reliability of this method among 11 sonic cephalometric measurements, 26 values demonstrated a weak correlation with the relative radiographic values [33]. Unfortunately, the patient’s actual clinical skeletal situation and the precise effect of therapy are still only evaluable by cephalometry. Every cephalometric analysis employs several anatomical skeletal points; some of these points are deep within the skull, such as the sella (S) or basion (Ba) points, and some are on the surface of the bone near the skin, such as the N, A, or B points. Deep points are often used to identify reference planes such as the ideal horizontal plane, despite the fact that the ability to accurately obtain this information in the natural head position rather than at the deep cephalometric points is still debated [31]. Superficial points on the bone are generally useful in representing the position of a whole skeletal structure; e.g., the A point represents the anterior limit of the whole maxilla, and the B point represents the anterior limit of the mandible. A complete cephalometric analysis is based upon both deep and superficial points; however, the skeletal maxillomandibular relations can only be examined by considering the superficial points. Even if the initial diagnosis requires comprehensive data including both deep and superficial points, it may be sufficient to limit the analysis to the superficial points when monitoring therapy progression. Steiner cephalometric analysis of the ANB angle, which only employs the surface points, well describes the maxillomandibular relations. When an orthopedic treatment is performed, a progression evaluation limited to improvement in the maxillomandibular relation may be sufficient to guide the orthodontist. The main aim of the present study was to identify the positions of the surface points of these bones to calculate the ANB angle without radiographs. Ultrasonographic evaluation of the ANB angle, which avoids radiographic exposure, may be repeated whenever necessary without any damage to the patient. In this study, we investigated the possibility of using ultrasonography to obtain an accurate measurement of the thickness of the soft tissues covering the N, A, and B cephalometric points. These measures may be employed to reconstruct the position of the underlying cephalometric points on a 1:1 photograph and thus calculate the ultrasonographic ANB angle.

## Materials and methods

**Fig. 1:**
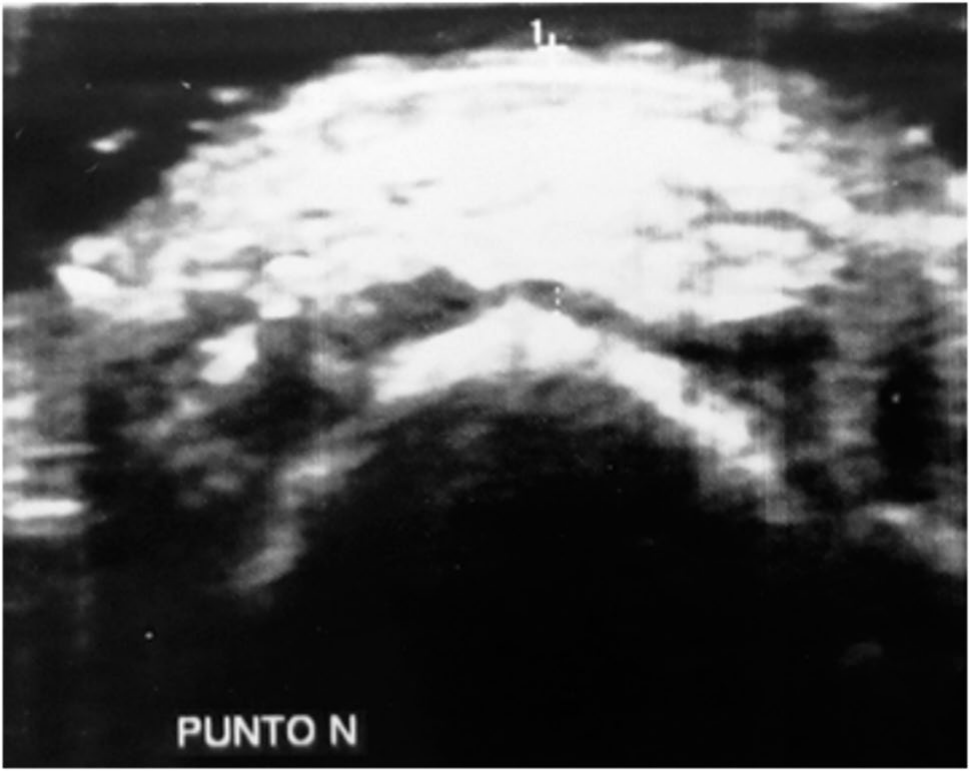
Ultrasonographic soft tissue thickness over N cephalometric point

**Fig. 2:**
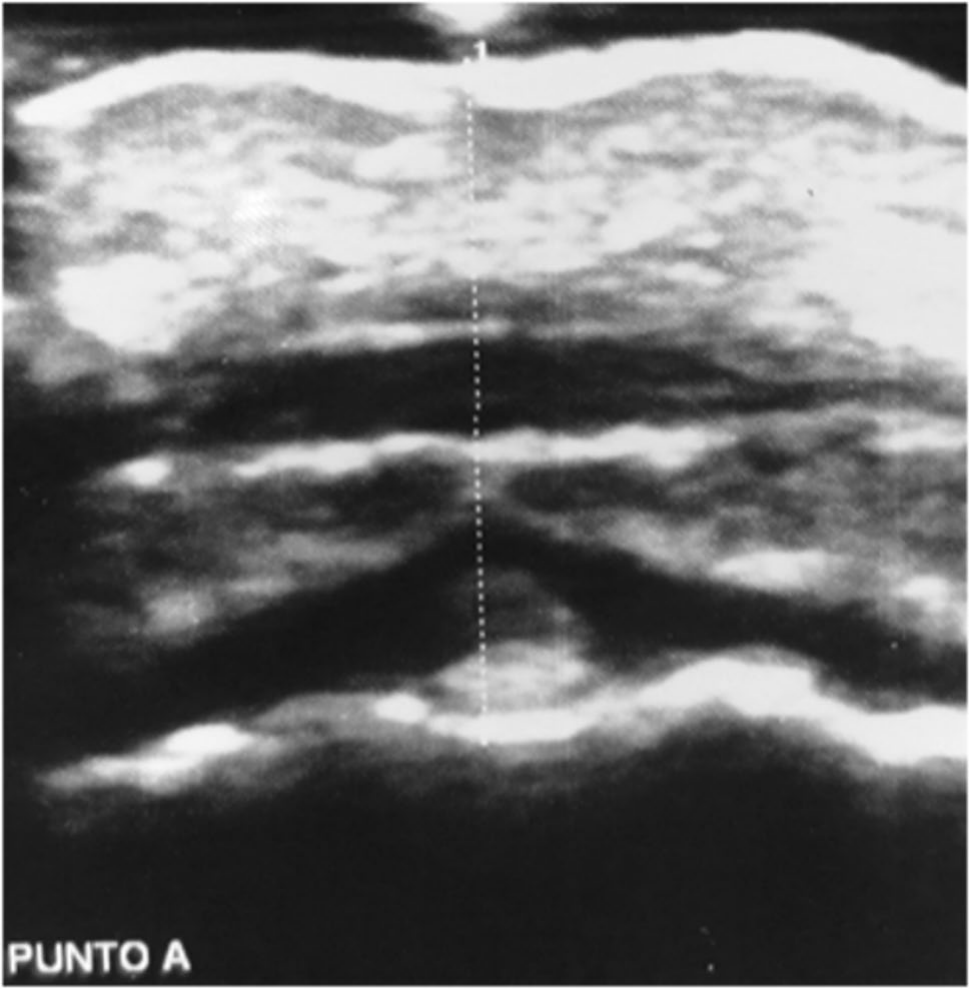
Ultrasonographic soft tissue thickness over A cephalometric point

**Fig. 3:**
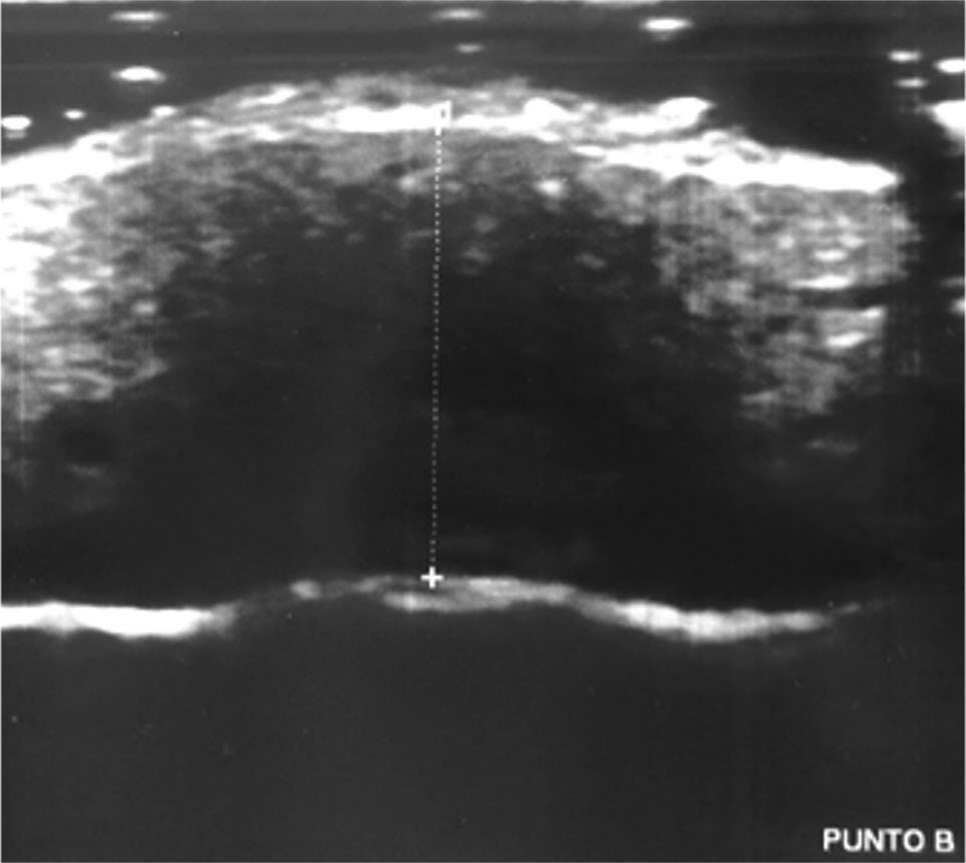
Ultrasonographic soft tissue thickness over B cephalometric point

**Fig. 4:**
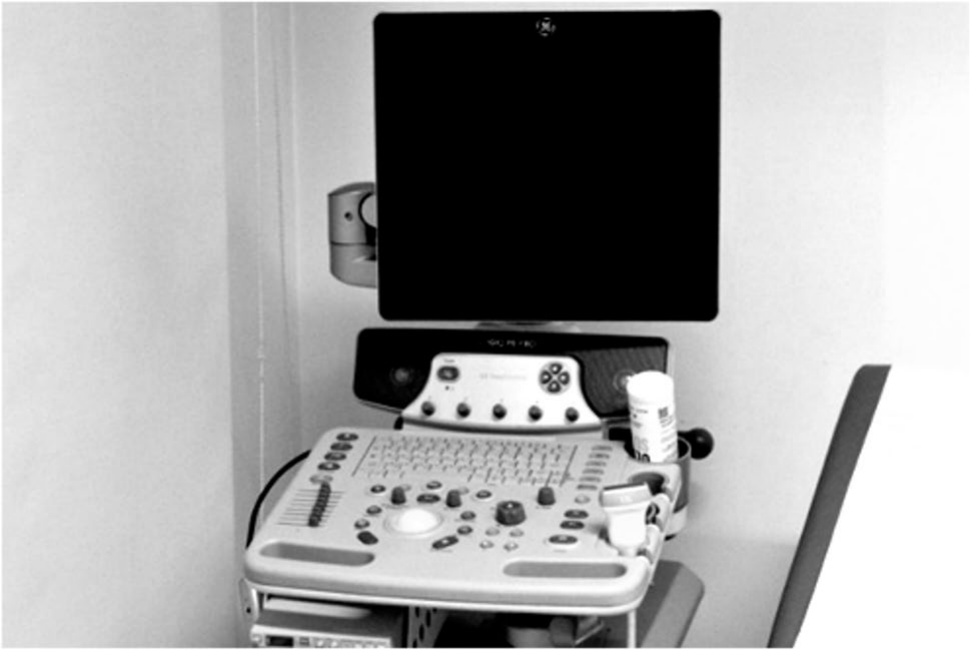
General electric LOGIQ P6 ultrasound system

**Fig. 5:**
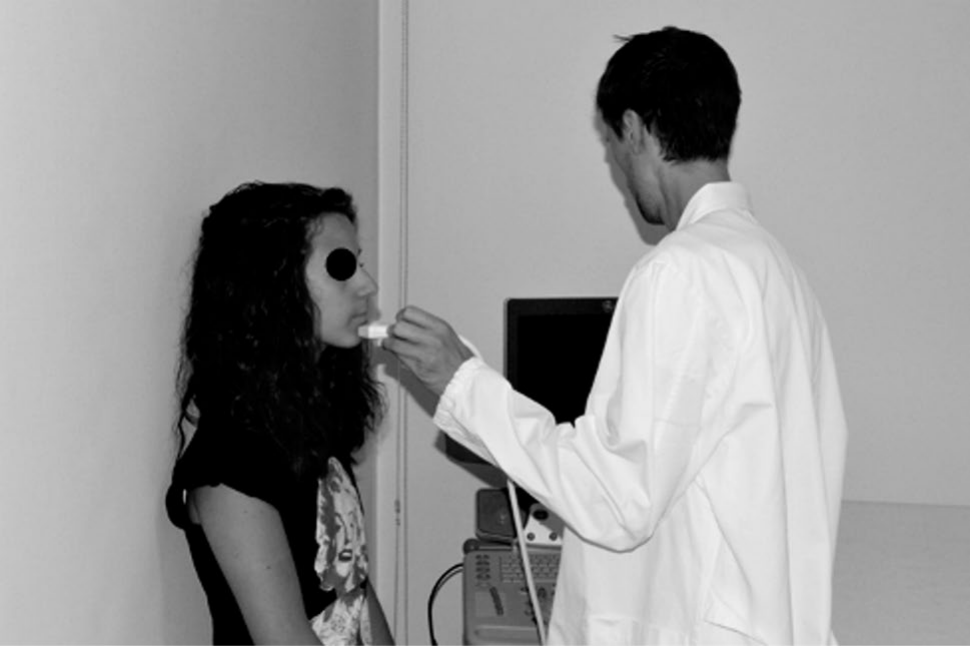
Ultrasonographic soft tissue thickness measurement

Twenty consecutive patients (9 male, 11 female; mean age 10.2 years; range 7.1–14.7 years) referred to a private practice in northern Italy for orthodontic treatment were evaluated in this study. The inclusion criterion was the presence of a digital lateral teleradiograph, obtained for orthodontic assessment of the maxillomandibular complex, that met the following requirements: the radiograph was obtained ≤30 days before the study, the patient’s occlusion was locked in a centric relation by an adequate amount of occlusal wax while the radiograph was taken, the lip posture was natural, and the radiograph was printed in a 1:1 ratio. Patients for whom orthodontic treatment had already been started and syndromic patients were excluded from the study. Radiographs were analyzed by two expert orthodontic tracers, who measured the Steiner ANB angle and thickness of the soft tissue covering the N, A, and B cephalometric points. The mean value between the measurements obtained by the two tracers was then employed in the study. A photographic image of the right profile of the face was then obtained with a Nikon D3200 camera (Nikon, Tokyo, Japan) with a Nikon Af-s 85-mm f/1.8 objective (Nikon) and a Bower SFD14C ring light flash (Bowen, New York, NY, USA). While taking the photograph, a ruler was maintained exactly in front of the midline of the facial profile and included in the photograph. This metric reference included in the photographs allowed for correction of the magnification when printing the photographs in a 1:1 ratio. Finally, ultrasonography was used to measure the thickness of the soft tissue above the N, A, and B cephalometric points (Figs. 1, 2, 3). The ultrasonographic data were collected with a General Electric LOGIQ P6 ultrasound system (Figs. 4, 5) with a linear transducer. The device setting was placed in the “small parts” position with a 13-MHz frequency, 20-mm penetration depth, 0.5 mechanical index, and 21-Hz/s fan rate. Both digital photographs and ultrasonographic data were collected while maintaining the patient in the same position as during the radiographic examination: standing up with the natural head position, occlusion in a centric relation, and a natural lip posture. Because of their characteristic depressions on the skeletal surface, the N, A, and B cephalometric points may be clearly identified by palpation. Their correct positions on the surface of the skin were identified in this way and marked by a small black dot. The ultrasonographic measurements were carried out by two expert operators. The operators reduced any possible bias during the ultrasonography by using a thick layer of coupling gel under the skin, avoiding direct pressure on the soft tissues; maintaining the ultrasound beam exactly on the black dots and perpendicular to the horizontal line (natural head position) to obtain high accuracy and reproducibility; and asking the patient to stop breathing for a few seconds when the ultrasonographic image was obtained.

**Fig. 6:**
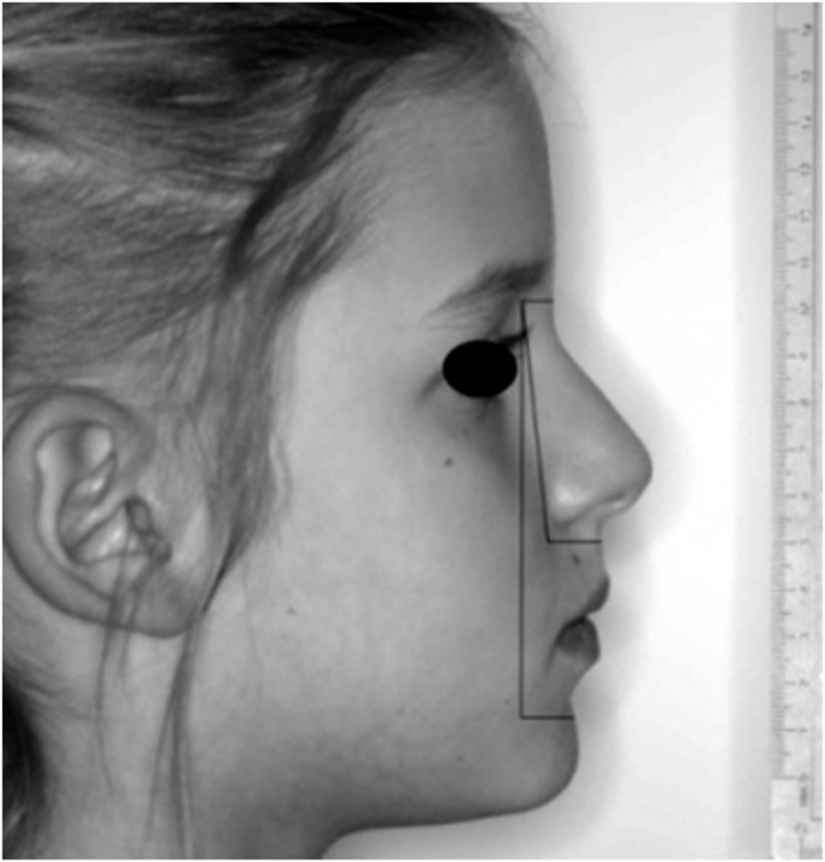
Ultrasonographic soft tissue thickness over N, A, and B cephalometric points marked on a 1:1 lateral photograph. ANB angle construction and measurement

The average of the measurements obtained by the two operators was marked on the 1:1 profile photographs to simulate the position of the relative skeletal cephalometric points. The “ultrasonographic/photographic” ANB angle was then calculated (Fig. 6). The following comparisons were considered: (1) average and single measurements of the N, A, and B points for the first versus second ultrasonographer; (2) average and single measurements of the ultrasonographic versus radiographic thickness of the soft tissues covering the N, A, and B points; and (3) average and single measurements of the ultrasonographic/photographic versus radiographic ANB angle.

## Results

Statistical analyses were performed with the software SPSS v.20 (IBM Corp., Armonk, NY), MedCalc v.12.5, and R v.3.0.2 using the packages epiR and irr. Mean and median differences between measurement series were evaluated by Student’s *t* test and Wilcoxon’s test, respectively. Overall effects were also evaluated by repeated-measures analysis of variance. Interoperator and intertechnique reliability was evaluated by Pearson’s correlation coefficient (*r*), the intraclass correlation coefficient, and Lin’s concordance correlation coefficient. Tukey–Bland–Altman plots were also compared (data not shown). Differences were considered statistically significant at *p* < 0.05.

### Single and average measurements of the N, A, and B points: first versus second ultrasonographer

Student’s *t* test and Wilcoxon’s signed-rank test showed no statistically significant difference between the mean soft tissue measurements overlying the N, A, and B points obtained by the two ultrasonographers (Table 1). Pearson’s and Spearman’s correlation coefficients as well as the intraclass and Lin’s concordance correlation coefficients between the measurements of the two operators are reported in Table 2. The various correlation coefficients show high reliability and concordance between the two ultrasonographers.

**Table 1:** Comparison of ultrasonographic measurements between the two ultrasonographers

Measurement point	Ultrasonographer 1, mean (SD) (mm)	Ultrasonographer 2, mean (SD) (mm)	Mean diff. (mm)	*p* value*
Point N	5.31 (1.14)	5.36 (1.01)	0.05	0.53
Point A	10.87 (0.98)	10.69 (0.98)	0.18	0.37
Point B	10.02 (1.40)	10.17 (1.25)	0.15	0.59

*SD* standard deviation, *diff*. difference

*Paired *t* test. Wilcoxon’s test (data not reported) provided similar results

**Table 2:** Correlation coefficients between the two ultrasonographers

	Pearson’s *r* (95% CI)	*r, p* value	Spearman’s *ρ* (95% CI)	*ρ, p* value	ICC (95% CI)	CCC (95% CI)
Ultra 1, N Ultra 2, N	**0.990** (0.949–0.998)	0.000	**0.983** (0.739 to 1.000)	0.000	**0.984** (0.939–0.996)	**0.982** (0.943–0.994)
Ultra 1, A Ultra 2, A	**0.837** (0.389–0.965)	0.005	**0.854** (0.303 to 1.000)	0.003	**0.838** (0.467–0.960)	**0.822** (0.408–0.955)
Ultra 1, B Ultra 2, B	**0.840** (0.397–0.965)	0.005	**0.672** (−0.106 to 0.987)	0.047	**0.844** (0.463–0.963)	**0.828** (0.433–0.956)

*Ultra 1* Ultrasonographer 1, *Ultra 2* Ultrasonographer 2, *ICC* intraclass correlation coefficient, *CCC* Lin’s concordance correlation coefficient, *CI* confidence interval

Bold values indicate the Pearson's and Spearman's correlation coefficients between the measurement of the two operators overlying the N, A and B points. There is also segnalated the intraclass correlation coefficient and the Lin's concordance correlation coefficients using 95% as the confidence interval

### Single and average measurements of thickness of soft tissues covering N, A, and B points: ultrasonographic versus radiographic results

**Fig. 7:**
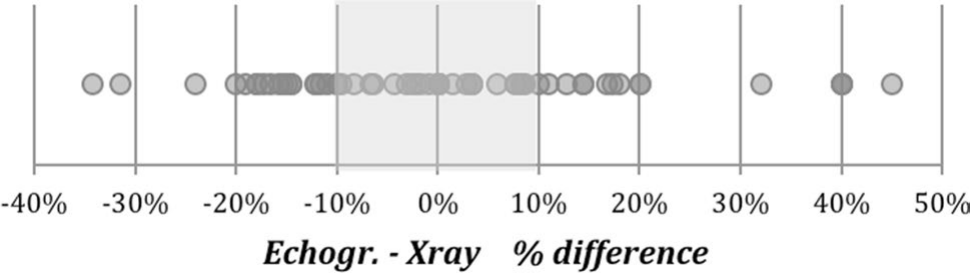
Most of the single measurements showed a difference of ≤20%

Student’s *t* test, the Mann–Whitney test, and mixed-model analysis of variance showed no statistically significant difference between the mean ultrasonographic and radiological measurements of the thickness of the soft tissues covering the N, A, and B points (Table 3). Comparison of the soft tissue thickness measured by ultrasound and X-ray revealed that most of the single measurements showed a difference of ≤20% (Fig. 7). However, only a weak correlation was found between these single measurements on the N, A, and B points (Table 4).

**Table 3:** Comparison of ultrasonographic versus radiographic measurements of the soft tissue thickness covering the N, A, and B bony points

Measurement point	Ultrasonography, mean (SD) (mm)	X-ray, mean (SD) (mm)	Mean diff. (mm)	*p* value*
Point N	5.33 (1.07)	5.58 (1.00)	0.24	0.55
Point A	10.78 (0.94)	11.22 (0.94)	0.44	0.24
Point B	10.09 (1.27)	9.39 (1.24)	0.70	0.20

*SD* standard deviation, *diff*. difference

*t test. Wilcoxon’s test (data not reported) provided similar results

**Table 4:** Correlation coefficients of ultrasonographic versus radiographic measurements of the soft tissue thickness covering the N, A, and B bony points

X-ray versus ultrasonography	Pearson’s *r* (95% CI)	*r, p* value	Spearman’s *ρ* (95% CI)	*ρ, p* value	ICC (95% CI)	CCC (95% CI)
Point N	**0.363** (−0.397 to 0.828)	0.336	**0.359** (−0.433 to 0.931)	0.343	**0.382** (−0.379 to 0.821)	**0.354** (−0.339 to 0.798)
Point A	**0.387** (−0.373 to 0.836)	0.316	**0.476** (−0.222 to 0.900)	0.195	**0.375** (−0.272 to 0.808)	**0.348** (−0.298 to 0.775)
Point B	**0.274** (−0.477 to 0.794)	0.472	**0.069** (−0.720 to 0.819)	0.861	**0.253** (−0.346 to 0.749)	**0.231** (−0.374 to 0.698)

*ICC* intraclass correlation coefficient, *CCC* Lin’s concordance correlation coefficient, *CI* confidence interval

Bold values indicate the Pearson's and Spearman's correlation coefficients between the measurement of the two operators overlying the N, A and B points. There is also segnalated the intraclass correlation coefficient and the Lin's concordance correlation coefficients using 95% as the confidence interval

### Single and average measurements of ANB angle: ultrasonographic/photographic versus radiographic results

Comparison of the average measurements for the ANB angle obtained by ultrasound and X-ray revealed no statistically significant difference (Table 5). Pearson’s and Spearman’s correlation coefficient as well as the intraclass and Lin’s concordance correlation coefficients showed a very strong correlation and concordance between the ANB angles obtained by ultrasound and X-ray (Table 6).

**Table 5:** Comparison of ultrasonographic versus radiographic measurements of the ANB angle

Measurement point	Ultrasonography, mean (SD) (mm)	X-ray, mean (SD) (mm)	Mean diff. (mm)	*p* value
ANB angle	6.00 (2.83)	6.06 (2.74)	0.06	0.76

Wilcoxon’s test (data not reported) provided similar results

*SD* standard deviation, *diff*. difference

**Table 6:** Correlation coefficients of ultrasonographic versus radiographic measurements of the ANB angle

X-ray versus ultrasonography	Pearson’s *r* (95% CI)	*r, p* value	Spearman *ρ*, (95% CI)	*ρ, p* value	ICC (95% CI)	CCC (95% CI)
ANB	**0.983** (0.916–0.996)	0.000	**0.962** (−0.730 to 1.000)	0.000	**0.984** (0.931–0.996)	**0.982** (0.924–0.996)

*ICC* intraclass correlation coefficient, *CCC* Lin’s concordance correlation coefficient, *CI* confidence interval

Bold values indicate the Pearson's and Spearman's correlation coefficients between the measurement of the two operators overlying the N, A and B points. There is also segnalated the intraclass correlation coefficient and the Lin's concordance correlation coefficients using 95% as the confidence interval

## Discussion

As stated in the introduction, only a complete cephalometric analysis provides a logical basis for treatment planning decisions. However, during the progression of a therapy, controlling the improvement in the maxillomandibular skeletal relation using a noninvasive method may be useful. The possibility of using ultrasonography to reliably measure the thickness of the soft tissues covering the N, A, and B cephalometric points was examined in the present study. No differences were found between the average measurements, but a very high correlation and concordance were found between the single measurements. The anatomical areas investigated in this study are very delicate and mobile. Any possible bias due to the risk of soft tissue compression by the ultrasound transducer or mobility of the soft tissues was evidently avoided by the employed method. If this was not the case, it would not have been possible to observe such large concordance and high correlation between the two operators. The ultrasonographic measurements were highly reproducible and not operator-dependent. The authors believe that this finding is very important because it creates the basis for future clinical use of the method.

The outcome of the comparison of the ultrasonographic and radiographic measurements of the soft tissue thickness covering the N, A, and B points is more difficult to interpret. Comparison of the means of the measurements demonstrated no statistically significant differences. Comparison of the single measurements, despite most showing a difference of ≤20% (Fig. 7), demonstrated only a weak correlation and concordance. The most probable explanation of this finding is that a 20% difference may be sufficient to justify the statistical outcome but irrelevant from the viewpoint of the whole ANB angle measurement. Further investigations involving larger sample sizes are in progress to better explain this observation regarding the single points.

The main objective of this study was to determine the reliability of ultrasonographic/photographic assessment of the ANB angle. The results of the statistical analysis seem very favorable and promising; in the authors’ opinion, these results overcome the previously reported statistical problem regarding a weak correlation and concordance between single-point measurements. In the present study, the observed difference between the single-point measurements did not modify the final value of the ANB angle. The ANB angle may be carefully measured by noninvasive ultrasonography in the clinical setting.

Combined photographic and ultrasonographic measurement of the ANB angle seems to provide the same data as the radiographic method. In this pilot study, the analysis was limited to the A, B, and N points to evaluate the feasibility and reliability of the proposed method. In the future, it will be possible to evaluate other profile contour points (i.e., Me and Pog), creating the basis for a more complete analysis.
